# Evidence-Based Caries Management for All Ages-Practical Guidelines

**DOI:** 10.3389/froh.2021.657518

**Published:** 2021-04-27

**Authors:** John D. B. Featherstone, Yasmi O. Crystal, Pamela Alston, Benjamin W. Chaffee, Sophie Doméjean, Peter Rechmann, Ling Zhan, Francisco Ramos-Gomez

**Affiliations:** ^1^Department of Preventive and Restorative Dental Sciences, School of Dentistry, University of California, San Francisco, San Francisco, CA, United States; ^2^Pediatric Dentistry Department, College of Dentistry, New York University, New York, NY, United States; ^3^Comprehensive Pediatric Dentistry, Bound Brook, NJ, United States; ^4^Department of Orofacial Sciences, School of Dentistry, University of California, San Francisco, San Francisco, CA, United States; ^5^Department of Operative Dentistry and Endodontics, UFR d'Odontologie de Clermont-Ferrand, Clermont-Ferrand, France; ^6^EA 4847, Clermont-Ferrand, France; ^7^Université Clermont Auvergne, Clermont-Ferrand, France; ^8^Service d'Odontologie, CHU Estaing, Clermont-Ferrand, France; ^9^Section of Pediatric Dentistry, School of Dentistry, University of California, Los Angeles, Los Angeles, CA, United States

**Keywords:** caries management, caries risk assessment, dental caries, fluoride, infants and toddlers

## Abstract

**Introduction:** The purpose of the present paper is to provide step-by-step guidelines for dental healthcare providers to manage dental caries based upon caries risk assessment (CRA) for ages 0–6 years and 6 years through adult. The manuscript reviews and updates the CAMBRA (caries management by risk assessment) system which includes CRA and caries management recommendations that are guided by the assessed risk level.

**Caries Risk Assessment:** CAMBRA CRA tools (CRAs) have been evaluated in several clinical outcomes studies and clinical trials. Updated CAMBRA CRAs for ages 0–6 years and 6 years through adult are provided. These CRAs have been refined by the addition of a quantitative method that will aid the health care provider in determining the caries risk of individuals.

**Caries Management Based Upon Risk Assessment:** Guidelines for individualized patient care are provided based upon the caries risk status, results of clinical exams and responses of the patient to questions in the CRA. These guidelines are based upon successful outcomes documented in several clinical outcomes studies and clinical trials. The paper includes a review of successful caries management procedures for children and adults as previously published, with additional emphasis on correct use of silver diamine fluoride (SDF) for children. The caries management plan for each individual is based upon reducing the caries risk factors and enhancing the protective factors with the additional aid of behavior modification. Beneficially altering the caries balance is coupled with minimal intervention restorative dentistry, if appropriate. These methods are appropriate for the management of dental caries in all patients.

## Introduction

Dental caries is a multifactorial, bacterially generated disease, where an unhealthy shift in the oral microbiome is driven by a diet that favors frequent ingestion of fermentable carbohydrates and behaviors, such as ineffective home oral hygiene practices, that allow the preservation of this unfavorable oral environment. In addition, other biological factors like salivary dysfunction or factors in the environment, like low health literacy or limited access to care, complicate the scenario; therefore, there is no single “magic bullet” that cures dental caries [1, 2]. The disease can be thought of as a balance between caries pathological and preventive factors as illustrated by the diagram in Tables 1, 2, [3–5]. The management of dental caries can be challenging when patients present with several caries risk factors. This is especially the case for patients with special needs.

**Table 1 (Part 1):**
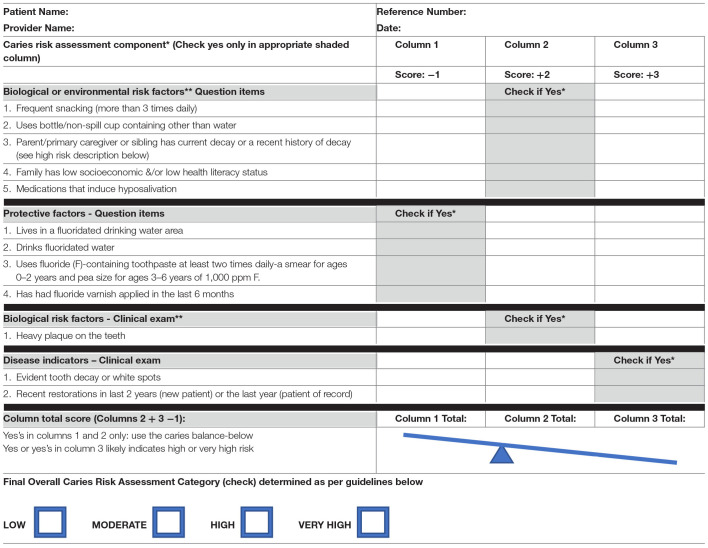
Updated CAMBRA Caries Risk Assessment form^#^ for ages 0–6 years (January 2021)^##^.

* *Check only the yes answers in the appropriate shaded column. Enter the score of −1, +2 or +3 for each yes checked. Unshaded columns are left blank. Assess the caries risk as per instructions in Table 1 (part 2) below*.

** *Biological and environmental risk factors are split into (a) question items, (b) clinical exam*.

# *Modified from Featherstone et al. [3] with permission of California Dental Association Journal*.

## *This material may be used free of charge for the purposes of patient care, education, academic works, research, health promotion, health policy and related activities. However, permission must be obtained before this material is used for commercial purposes*.

*Copyright © 2003, 2007, 2010, 2011, 2019, 2020, 2021 The Regents of The University of California. CAMBRA is a trademark of the Regents of The University of California. Except where otherwise noted, this content is licensed under a Creative Commons 4.0 International license (CC BY-NC-ND 4.0)*.

*Refer to the second page of this form (part 2) for instructions for use as guidelines for caries risk assessment*.

**Table 1 (Part 2):** Caries risk assessment guidelines 0–6 years.

The dental caregiver has the responsibility of making a caries risk assessment and then deciding on a caries management plan for the patient that leads from the risk assessment and a personalized assessment of the needs of the individual patient. These guidelines can assist in the process. **Determining the caries risk as low, moderate, high or very high - guiding principles.** 1. Low risk. If there are protective factors, very few or no risk factors, no disease indicators, and the protective factors prevail, the patient is at low risk. 2. Moderate risk. If there are no disease indicators and the risk factors and protective factors appear to be balanced then a moderate caries risk determination is appropriate. If in doubt move the moderate to a high classification. 3. High risk. If there is a “YES” in column 3 (one or both disease indicators) the patient is very likely at high risk. Even if there are no “yes” disease indicators the patient can still be at high risk if the risk factors definitively outweigh the protective factors. Parent or caregiver with current or recent dental decay most likely indicates high caries risk for the child. 4. Very high risk. If the above process indicates high risk and the existing or recent decay is severe and/or extensive a designation of “very high” caries risk is appropriate and will guide a more aggressive caries management plan. Any items checked “yes” should also be used as topics to modify behavior or determine additional therapy.
**Use the following modified caries balance** to visualize the overall result and determine the risk level. It may be helpful to allocate scores for each “yes” checked on the risk assessment form with a score of −1 for yes's in column 1, and +2 and +3 respectively for yes's in columns 2 and 3. The final total will help guide the risk level decision. **Low** = –4 to –1; **Moderate** = 0 to +3; **High** = +4 to +13; **Very high** = +14 to +18 and/or is a high risk level plus extensive and/or severe recent or existing decay.
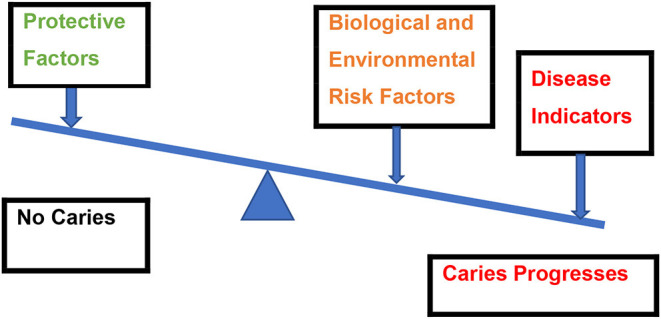
**Additional caries-related components for caries management and caregiver/patient counseling.** **Record in patient chart at each visit**.
Dietary counseling to reduce frequency and amount of fermentable carbohydrates, especially sucrose, fructose (high fructose corn syrup) and continual fruit juice (e.g., apple juice). Record number and type of daily snacks, drinks and juices used.
Bottle used continually, bottle used in bed or nursing on demand. Record details provided.
Fluoride (F) toothpaste use. Note frequency and amount used at each visit.
Record all recommended therapy such as F toothpaste, F varnish, use of silver diamine fluoride in appropriate cases. Record usage provided by parent/caregiver.
Record medications at each visit and check for changes.
Record participation in assistance programs such as “school lunches,” “head start,” appropriate to the state or country.
Child has developmental problems/child has special care needs (CHSCN).
Inadequate saliva flow and related medications, medical conditions, or illnesses.
**Discuss self-management goals with caregiver/patient and set two goals together at each visit. Provide in writing**.

**Table 2 (Part 1):**
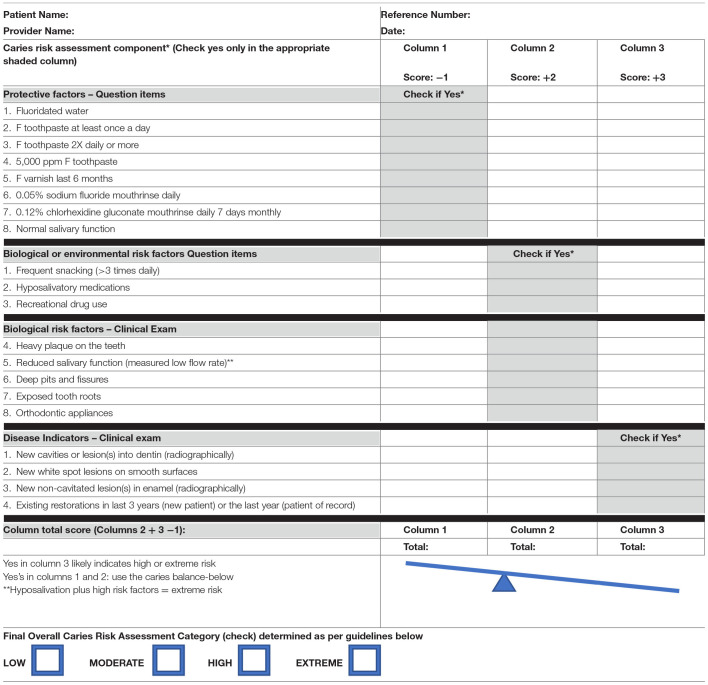
Updated CAMBRA Caries Risk Assessment form^#^ for ages 6 year through adult (January 2021)^##^.

* *Check only the yes answers in the appropriate shaded column. Enter a score of −1, +2 or +3 for each yes checked. Unshaded columns are left blank. Assess the caries risk as per instructions in Table 2 (part 2) below*.

** *Hyposalivation plus high risk factors = extreme risk*.

# *Modified from Featherstone et al. [4] with permission of California Dental Association Journal*.

## *This material may be used free of charge for the purposes of patient care, education, academic works, research, health promotion, health policy and related activities. However, permission must be obtained before this material is used for commercial purposes*.

*Copyright © 2003, 2007, 2010, 2011, 2019, 2020, 2021 The Regents of The University of California. CAMBRA is a trademark of the Regents of The University of California. Except where otherwise noted, this content is licensed under a Creative Commons 4.0 International license (CC BY-NC-ND 4.0)*.

*Refer to the second page (part 2) for instructions for use as guidelines for caries risk assessment*.

**Table 2 (Part 2):** Caries Risk Assessment Guidelines for ages 6 years through adult.

The dental caregiver has the responsibility of making a caries risk assessment and then deciding on a caries management plan for the patient that leads from the risk assessment and a personalized assessment of the needs of the individual patient. These guidelines can assist in the process. **Determining the caries risk as low, moderate, high or extreme - guiding principles** 1. **Low risk**. If there are no disease indicators, very few or no risk factors and the protective factors prevail, the patient is most likely at low risk. Usually this is obvious. 2. **Moderate risk**. If the patient is not obviously at high, or extreme risk and there is doubt about low risk, then the patient should be allocated to moderate risk and followed carefully, with additional chemical therapy added. An example would be a patient who had a root canal as a result of caries 4 years ago, and has no new clinical caries lesions, but has exposed tooth roots and only uses a fluoride toothpaste once a day. 3. **High and extreme risk**. One or more disease indicators most likely signals at least high risk. If there is also hyposalivation the patient is likely at extreme risk. Even if there are no positive disease indicators the patient can still be at high risk if the risk factors definitively outweigh the protective factors. Think of the caries balance: visualize the balance diagram as illustrated below. Any items checked “yes” should also be used as topics to modify behavior or determine additional therapy.
**Use the following modified caries balance** to visualize the overall result and determine the risk level. It may be helpful to allocate scores for each “yes” checked on the risk assessment form with a score of −1 for yes's in column 1, and +2 and +3 respectively for yes's in columns 2 and 3. The final total will help guide the risk level decision. **Low** = −8 to −2; **Moderate** = −1 to +2; **High** = +3 to +17; **Extreme** = +18 to +30 and/or is a high risk level plus measured or observed hyposalivation. Use the caries balance to visualize the overall result and determine the risk level for the individual patient.
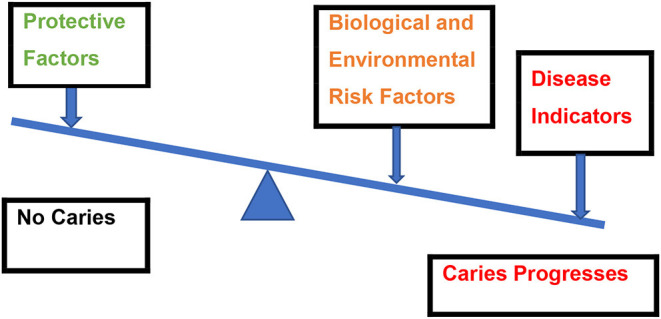
**Additional caries-related components for caries management and caregiver/patient counseling.** **Record in patient chart at each visit**.
Dietary counseling to reduce frequency and amount of fermentable carbohydrates. Record number and type of daily snacks, drinks and juices used.
Oral hygiene and fluoride (F) toothpaste use. At each visit note frequency and amount used.
Record all recommended therapy such as F toothpaste, F varnish, chlorhexidine and usage by patient.
Record medications at each visit and check for changes.
Record participation in assistance programs such as “school lunches,” “head start,” appropriate to the state or country.
Child or adult has developmental problems or special care needs (CHSCN).
Inadequate saliva flow and related medications, medical conditions, or illnesses.
**Discuss self-management goals with caregiver/patient and set two goals together at each visit. Provide in writing**.

Dental caries may be managed by swinging the caries balance toward the protective factor side and maintaining it there by reducing pathological risk factors and promoting protective factors [6]. It is now well-recognized that caries management is best done on a personalized basis building upon a reliable caries risk assessment (CRA) where detailed information about the specific risk factors of a patient can be utilized not only to establish the risk of developing future caries lesions, but also to establish an effective plan to promote protective habits with the aid of behavior modification and to tailor the periodicity of oral evaluations. Assessment of caries risk for each individual patient is essential as the basis for the management of dental caries for patients of all ages [7, 8].

The procedures and philosophy known as “caries management by risk assessment” and abbreviated to CAMBRA® were published in the Journal of the California Dental Association in 2007 and updated in 2019 for patients aged 6 years through adult [4, 9, 10], as well as for young children aged 0–5 years [3, 11] and have been utilized for over 15 years in the teaching clinics of the School of Dentistry at the University of California San Francisco (UCSF) [12] and at the University of California Los Angeles (UCLA) School of Dentistry Pediatric Dental Clinic, as well as several community health centers in California [13, 14]. Successful management of dental caries requires (a) the use of a reliable CRA tool, that then leads to (b) the formulation of an individualized treatment plan that is derived from the caries risk level and the information learned during the CRA process. The overall CAMBRA method includes both risk assessment and caries management.

The CAMBRA CRA tool was developed over decades by personnel at UCSF as described above, based upon research on key factors that contribute to caries progression or reversal on real patients over time. The tool was launched in 2003 and has been updated since then based upon clinical outcomes [3, 4, 10, 11, 15]. It provides a risk assessment form for two age ranges, namely ages 0–6 and 6 years through adult. The CAMBRA CRA tool has been shown to be highly predictive of future caries lesions in three different studies, totaling more than 20,000 patients, for the age group 6 years through adult and for the age group 0–5 years [12, 16–18]. A detailed discussion of these clinical studies has been reported previously [3, 4]. CAMBRA CRA can confidently be used by the dental care provider to assess the caries risk of an individual patient and to use the risk level as a basis for developing a caries management plan.

The purpose of the present paper is to provide step-by-step guidelines for dental healthcare providers to manage dental caries based upon CRA for all ages. The manuscript reviews and updates the CAMBRA system which includes CRA and caries management recommendations that are guided by the assessed risk level [3, 4, 6, 9, 11, 12, 15, 19–21]. In this paper, we include the use of a quantitative component with the CAMBRA CRA forms to aid the clinician in the determination of the caries risk level.

## Caries Management Based Upon Risk Assessment—Practical Guidelines for the Health Care Provider

Several segments of this publication are reproduced or modified from Featherstone et al. [3, 4], Rechmann et al. [22], and CAMBRA guide [23] with permission of the publishers.

### Definitions of Terminology for Caries Risk Assessment

In the present publication caries risk, risk factors, protective factors and disease indicators are defined as follows:

a) ***Caries risk*** is the likelihood of the patient having new caries lesions (active white spots, non-cavitated approximal lesions, cavitated lesions) in the near future.
b) ***Protective factors*** are environmental factors, biological factors or chemical therapy that help to swing the caries balance to caries lesion prevention or reversal. Examples are fluoride in drinking water, adequate saliva and the use of fluoride toothpaste.
c) ***Risk factors*** are environmental or biological factors that contribute to the initiation or progression of caries lesions. Biological factors include items such as acid producing bacteria, visible plaque on the teeth, frequent snacking on fermentable carbohydrates. Environmental factors include items such as low health literacy (Tables 1, 2).
d) ***Disease indicators*** are the clinically observed results of previous and/or ongoing dental caries destruction of the tooth mineral. They do not contribute to the disease, but they are direct indicators of the presence of disease in the past or at the time of the observation.
e) ***Caries lesion*. **In this publication the term “caries lesion” is used throughout to describe a dental lesion (cavitated or non-cavitated) caused by the dental caries process. The term caries lesion may also be referred to as a carious lesion.

### General Considerations for Successful Caries Management

Assessment of the caries risk level for future occurrence of caries lesions is an important first step in managing dental caries and monitoring oral health improvement over time. All children should be given their first oral exam upon the eruption of the first tooth or before 1 year of age to ensure early intervention and prioritize prevention over restoration. Successful management of dental caries requires a risk-based approach to formulate an individualized treatment plan using a chronic disease management model, which aims at targeting the specific biological and environmental risk factors (environmental includes social) that contribute to the establishment and progression of this multifactorial disease. This individualized treatment plan should include behavior modification (for diet improvement, less sugar intake, plaque control, adherence to use of prescribed products) and non-surgical caries management [20, 21], in addition to appropriate minimally invasive restorative treatment, if required. The caries risk level determines the personalized caries management approach for each individual patient. As stated by Ramos-Gomez and Ng [24] “Since the risk for caries development and caries activity differs among individuals and may change in each individual over time, CRA performed initially, and periodically thereafter, allows for a determination of a patient's relative risk, from which is developed an evidence-based prevention plan that can be customized.”

Personalization further takes into consideration the behavioral barriers of the individual child or adult and the social context of the child/family/individual. It is very important to emphasize that the use of the CAMBRA tool for young children is a unique way to establish trust with the parent/caregiver by addressing the “risk factors” first, as a way to ease into a non-judgmental conversation and dialogue. In the care of infants and toddlers, it is essential to recognize our role as “health coaches and behavioral interventionists” when talking with parents/caregivers, in order to introduce good positive oral health behaviors at home in their daily living. The way in which this is done may vary according to the social environment and the individual culture of the patients and/or families involved.

The caries risk level is determined by the health care provider as low, moderate, high or very high/extreme by visualizing the “caries balance” as described above to weigh the preventive factors, biological and environmental risk factors and the disease indicators (clinical observations) and finally the clinical judgment of the care provider (Tables 1, 2). Step-by-step CRA procedures for age groups 0–6 and 6 years through adult are provided below. The practitioner will decide which version is appropriate to use for each individual patient. In this review we have updated the CAMBRA CRA tools (CRAs) for each age group as summarized in Tables 1, 2.

In these latest CRA tools only risk assessment components that have been proven to be significantly related to ongoing caries in clinical outcomes studies are included. Further, several modifications have been made in order to make the forms more user friendly. The layout of the CRA forms (Tables 1, 2) has been restructured so that protective factors, risk factors and disease indicators are listed from left to right to indicate that protection and risk reduction are paramount and to match the caries balance concept more clearly. The order of the items in the Tables are arranged so that non-clinical questions can be answered first prior to a clinical examination. We have also incorporated a simple quantitative method to the CAMBRA forms (termed CAMBRA 123) that helps visualize the caries balance more effectively and aids the oral health care providers in their determination of risk level. Precise instructions are provided how to use this on the face of each form and in more detail in part 2 of each of the two forms (Tables 1, 2).

The final determination of the caries risk level lies with the health care provider, based upon validated risk assessment guidelines coupled with other factors observed by the practitioner and his/her clinical judgment.

The following sections that present guidelines for CRA and caries management are designed to stand alone for each of the two age groups, namely 0–6 and 6 years through adult. There is some necessary repetition in order for each section to be used as a stand-alone document.

### Caries Risk Assessment–Practical Step-By-Step Guidelines for the Age Group 0–6 Years

Commencing a CRA is the first of six steps of an oral care visit for ages 0–6 years. These six steps include:

1. CRA (CAMBRA) is initiated and is subsequently completed in step 5 below
2. Knee to knee exam
3. Toothbrush prophylaxis
4. Clinical examination
5. Determine the caries risk level. Develop a caries management plan (CAMBRA) based upon the caries risk level, clinical observations, answers to questions, etc., as described in section Caries Management Based on Risk Assessment- Practical Step-By Step Guidelines for the Age Group 0–6 Years below (may, for example, include such things as a fluoride varnish application)
6. Self-management goals (anticipatory guidance) [24].

This section focuses on the CRA procedure. Parts of the following sections are reproduced and updated with permission from Featherstone et al. [3].

The following are step-by-step guidelines for use of the CAMBRA CRA tool with young children ages 0–6 years. The updated CRA procedure for the age group 0–6 years (Table 1) identifies low, moderate, high and very high risk for this age group. CRA takes place as part of the regular comprehensive or periodical oral exam in the following sequence, or in a sequence that suits the workflow of each individual practice or practitioner. The questions in the CRA can be answered initially by the parent/caregiver, or in conjunction with a dental assistant, hygienist or other staff member prior to the clinician seeing the patient and parent/caregiver together. CRA is the basis for formulating an individualized caries management treatment plan as described in detail below. Here are the steps in the CAMBRA CRA process for the 0–6 year age group:

1. From the medical, dental and social histories reported, compile relevant data to record in the CRA form (Table 1, columns 1 and 2).
2. Talk to the parent or other caregiver to make sure all questions listed in the CRA form are answered (Table 1, columns 1 and 2). The discussion will include details of the risk factors and protective factors, leading to the subsequent clinical exam and later to a discussion of self-management goals. This step is purposely done before the clinical exam of the child.
3. Conduct the clinical examination in an age-appropriate way: knee to knee or with child sitting on his/her own, ideally with the parent being able to be shown the findings. Start with detecting and recording presence of plaque, ideally with visible plaque index score (VPI), and showing the parents the problem areas. This answers the heavy plaque question in Table 1, column 2. Follow with a toothbrush prophy to remove debris and clean surfaces for better visualization during the exam, showing the parents the proper brushing technique [24]. The use of a flosser for interdental plaque removal, when appropriate, should also be demonstrated.
4. From the intra-oral examination detect and record caries lesions from their earliest (white spots, which can be arrested or reversed by remineralization) to their most advanced (cavitated) stages. From radiographical bitewing examination (if available depending on child's age and cooperation and local regulations), detect and record radiographic decay. This completes the disease indicator section of Table 1, column 3.
5. Assess and document the caries risk level as low, moderate, high or very high. It is the responsibility of the dental care provider to make the final judgment of caries risk status based upon the data collected on the CRA, taking into consideration other factors like expected parental compliance to recommendations and re-care visits, coupled with the provider's clinical judgment. Apply fluoride varnish if appropriate.

Steps 1, 3 and 4 are familiar elements of any conventional oral examination for this age group. Step 2 compiles a few simple questions (as listed in the CRA form in Table 1, columns 1 and 2) that attempt to identify the potential causes of the ongoing disease or to evaluate whether it is under control. Only those biological risk factors that have been shown to be statistically significantly related to ongoing caries and successful risk assessment in previous studies are included here [17, 25]. Table 1 is a ready to use CRA form that provides a visual summary of the factors that contribute to the overall caries risk assignment. Definitions of terms and justification for inclusion are.

#### Biological and Environmental Risk Factors—Table 1, Column 2

Biological risk factors contribute directly to the initiation or progression of dental caries (both the caries disease and caries lesions). They include an assessment of factors that have been established as most important (Table 1). The risk factors utilized in this CRA form are:

1. Frequent snacking on fermentable carbohydrates, at least three times daily outside of mealtimes.
  Frequent carbohydrate intake results in a prolonged acidic environment in the plaque that dissolves the tooth mineral and can act as a driving force to reinforce the overgrowth of cariogenic bacteria and the suppression of oral commensal (beneficial) bacteria, leading to future caries development [26]. Fermentable carbohydrates such as sucrose, fructose (high fructose corn syrup), glucose, and cooked starch are included. Fruit juice (e.g., apple juice) is an important but often overlooked source of fermentable carbohydrates among young children.
2. Use of bottle or non-spill cup containing liquids other than water.
  This provides a continuous ingestion of carbohydrates, such as from fruit juices, that leads to a continual acid environment in the plaque. It should be stressed that the use of milk in a bottle overnight and/or nursing on demand in the presence of cariogenic bacteria provides a prolonged acid challenge that increases the risk for caries and should be strongly discouraged.
3. Mother/primary caregiver or sibling has current decay or a recent history of decay.
  Presence of recent decay indicates they have high levels of cariogenic bacteria, especially Mutans Streptococci (MS), that can be transmitted to the child. Early colonization of MS by 3 years of age will increase the child's risk for developing caries [26, 27]. Current or recent decay in the parent or caregiver is an important indicator of potential high caries risk for the child. This becomes more important in infants with few teeth present, where signs of additional risk factors are not yet evident, and is supported by the strong correlation found in numerous studies [28–31].
4. Family has low socioeconomic and/or low health literacy status.
  Low socioeconomic status, of course, is not a biological contributor to the caries process. However, as a social determinant of health for many other diseases, it is one of several statistically significant factors associated with high caries risk [17, 25]. Practitioners should account for a challenging family socioeconomic context in formulating a personalized caries management plan. Similarly, low health literacy is not a biological risk factor, but it is often associated with socioeconomic level and contributes to increased risk of disease. Importantly, it is possible to educate the parent/primary caregiver regarding caries and its prevention.
5. Use of medications that induce hyposalivation.
  Hyposalivation is a side effect of some of the most commonly prescribed medications such as those used to treat allergies, asthma, mental disorders and cancer [32]. The risk of dry mouth increases with the number of medications prescribed. Hyposalivation can also be caused by other factors including some medical conditions and genetic factors.

**In the CRA procedure**, any items on this list with a positive response are marked with a yes (Table 1, column 2). Each yes adds to the risk level. Items 1 and 2 can be modified by behavioral management. A yes to item 3 may indicate a potentially very high risk patient that requires additional care and therapy.

#### Biological Risk Factors—Clinical Exam—Table 1, Column 2

##### Heavy Plaque on the Teeth

This simple measure, as observed by the clinician, has been shown in our clinical outcomes studies in children of all ages and in adults, to be a strong indicator of cariogenic bacterial activity, and it is strongly related to ongoing caries [12, 17, 18, 25]. This factor may indicate a combination of items that include high levels of cariogenic bacteria, ineffective plaque removal, food accumulation, and inadequate brushing with fluoride toothpaste. Gingivitis, or gums that bleed easily can be a sign of consistent presence of heavy plaque in specific areas, and a clinical risk indicator related to presence of plaque.

There is ample evidence that cariogenic bacteria levels are strongly related to caries risk [33–36]. However, at the time of writing there is no validated chairside test commercially available for measuring cariogenic bacterial levels. Therefore, cariogenic bacteria counts have been eliminated from the CRA form in this revised version. A quantitative bacteria test can be added back at a later date when an evidence-based chairside test becomes available.

**In the CRA procedure**, any items on this list with a positive response are marked with a yes (Table 1, column 2). Each yes adds to the risk level. Heavy plaque on the teeth can be modified by behavioral management.

#### Protective Factors - Table 1, Column 1

Protective factors are biological factors, environmental factors or chemical therapy that help to swing the caries balance to caries lesion prevention or reversal (Table 1, part 2). The factors included in the 0–6 years age group CAMBRA CRA form are:

1. Lives in a ***fluoridated drinking water area***
2. ***Drinks fluoridated water***
  The beneficial effect of drinking fluoridated water is well-established.
3. ***Uses a fluoride-containing toothpaste at least twice daily***
  The beneficial effect of brushing with fluoridated toothpaste has been well-established in numerous clinical trials and is a major factor in reductions in caries over recent decades [37–40]. The American Academy of Pediatric Dentistry (AAPD) and the American Dental Association (ADA) recommend at least twice-a-day use of a smear of a fluoride toothpaste for ages 0–2 years and a pea size for ages 3–6 years. when using a 1,000 part per million fluoride (ppm F) toothpaste [38, 41]. For children ages 0–6 years, it is recommended that the parent/caregiver brushes the child's teeth, or supervises toothbrushing, twice a day. Parent-supervised toothbrushing with F toothpaste (preferably 1,000 ppm F or higher) at least twice daily provides considerable added benefit above once daily [42, 43]. Countries and regions other than USA have published different guidelines appropriate to the region.
4. Has had ***fluoride varnish*** applied in the last 6 months
  The caries-reducing benefit of fluoride varnish (FV) is well-established, including when used in young children [44, 45].

**In the CRA procedure** each of these items with a positive response receives a “yes” score in column 1, Table 1.

Note: xylitol use by the caregiver is no longer listed as a protective factor in this revised CRA version as the evidence of its antimicrobial effects to achieve caries prevention is limited for adults or children [46]. However, xylitol is non-cariogenic and its use is still recommended to substitute other sugars to reduce frequency of snacking on fermentable carbohydrates [46].

#### Disease Indicators – Clinical Exam—Table 1, Column 3

**Disease indicators** are the clinically observed results of previous and/or ongoing dental caries destruction of the tooth mineral. They do not contribute to the disease; they are simply manifestations and clinical signs of the effects of dental caries at different stages. Disease indicators fit into two overall descriptions as evaluated in the outcomes assessments over several years of the original CAMBRA CRA form for the 0–6 year age group. They are strong indicators of ongoing disease.

1. Evident tooth decay or white spots
  This descriptor includes:
  a) Observed cavitation or radiographic evidence of progression into dentin,
  b) White spot lesions (that are new or active) on smooth surfaces,
  c) Radiographic or visual evidence of non-cavitated demineralization into the enamel (usually by bitewing radiographs).
2. Existing restorations
  Restorations that were placed due to caries in the last 2 years for a new patient or the last year for a patient of record. For a new patient visit, one or more of these disease indicators signals “high caries risk.” For a patient of record at a follow up visit any new appearance of tooth decay, white spots, or recent restorations signals “high caries risk.” If hyposalivation is present, in addition, this will require additional care and therapy.

#### Determination of Caries Risk (Table 1)

Details are provided in part 2 of Table 1. In addition to the written guidelines the determination of caries risk level is guided by visualizing the caries balance from the results on the CRA form or when using an electronic version of the questions and clinical observations. To aid in this visualization we have included a simple quantitative tool known as CAMBRA123. Protective factors in column 1 that are marked yes each receive a score of −1. Risk factors in column 2 with a yes are each scored +2. Yes to disease indicators in column 3 each receive a score of +3. Then simply add the scores for columns 2 and 3 and subtract the total from column 1. Consult the chart in Table 1, part 2 and be guided to a caries risk level.

#### Oral Health During Pregnancy and Maternal Pre-natal Caries Risk

Because maternal prenatal oral health is linked to the oral health of the child, it is necessary to address the maternal prenatal risk factors for caries in children and the possibility of caries transmission from mother to child [47, 48]. Emphasizing early interventions for women during pregnancy is recommended to improve the likelihood of early intervention for the child. Although misconceptions still exist regarding the safety and effectiveness of oral health care for pregnant women, in reality the establishment of a healthy oral environment for pregnant women is both important and achievable, and includes plaque control through brushing, flossing, use of F toothpaste and antimicrobial agents (e.g., chlorhexidine rinses). This can be followed by a professional prophylaxis including coronal scaling, root planning, and polishing. Expectant mothers should be encouraged to continue these practices after the child is born as a means of promoting oral health for the mother and her infant [47].

### Caries Management Based on Risk Assessment- Practical Step-By-Step Guidelines for the Age Group 0–6 Years

The following are step-by-step guidelines for use of the CAMBRA system for caries management with young children ages 0–6 years. Parts of the following sections are reproduced and updated with permission from Featherstone et al. [3].

1. Carry out a CRA as described above and classify the child as low, moderate, high or very high caries risk.
2. Produce and document a caries management plan that addresses all the risk factors that may contribute to the development or progression of disease for that specific patient, including lifestyle/behavior modification for caregivers and child to achieve plaque control and diet improvements [24].
3. Prescribe and/or provide chemical therapy for the patient, that includes fluoride with or without antibacterial therapy, based upon the caries risk level and the age of the patient. Details are described below. Provide anticipatory guidance and integrate motivational interviewing principles for caregivers and patients (when age appropriate) to set up achievable self-management goals for home management plans [20, 21, 49].
4. Develop a restorative treatment plan (if necessary) that takes into consideration age, behavior (cooperation for treatment delivery), health status and social determinants, favoring minimally invasive restorative procedures to conserve tooth structure whenever possible, restoring function and aiming at providing that patient with the means to achieve adequate plaque control.
5. Establish periodicity of recalls, and review at intervals appropriate to the caries risk status, to continue active surveillance of non-cavitated lesions, provide in-office preventive measures, and reinforce behavioral changes and adherence to prescribed daily home regimes.
6. Reassess and document caries risk level at each recall and modify the caries management plan and self-management goals as necessary.

CAMBRA therapies for older children and adults place special importance on chemical therapy, because placing restorations can restore tooth form and function but does not affect the risk factors that caused the disease, such as a cariogenic diet or high levels of cariogenic bacteria in the rest of the mouth [50–52]. One recommended antimicrobial chemical therapy in children 6 years and older and in adults as part of a caries management plan is chlorhexidine mouthrinse [12, 50]. However, use of chemotherapeutic agents in infants and toddlers requires special considerations due to toxicity/safety and behavioral acceptance issues. For this reason, in this age group, most of the recommendations within a caries management plan rely heavily on a chronic disease management model, where different strategies, such as education about the disease process, motivational interviewing style counseling (to change diet practices and plaque control routines), and periodic evaluation of self-management goals in conjunction with age appropriate chemical therapy to modify the oral pH environment, are used to target the individual risk factors that can trigger the disease process on the individual patient (frequent snacking, bottle feeding, visible plaque accumulation, etc.) [11, 20, 21, 24]. Several publications describe in detail this style of counseling and surveillance [14, 15, 20, 21, 24].

When addressing oral health in high risk groups, early intervention and strategic disease management are key. The importance of early assessment, diagnosis, and intervention as a means of oral disease prevention management must be stressed [14, 15, 20, 21, 24]. Early intervention and education are the most effective ways to prevent problems that traditional infectious-disease models fail to address. Advocacy and promotion of an age-one visit is critical in preventing early childhood caries and laying a foundation of good oral health throughout the life course [24]. All children should receive their first oral exam upon the eruption of their first tooth or before 1 year of age.

In evidence-based minimum intervention dentistry, which includes use of CAMBRA, fluoride, sealants (preventive and therapeutic), remineralization substances such as casein phosphopeptide, prevention of early cariogenic bacteria colonization by xylitol product use for family members with caries, and acid neutralization agents such as baking soda wiping after meal/snacks, the patient/caregiver is encouraged to assume responsibility for the level of infection and is educated, instructed, and monitored in the proper control techniques. It is the child who has the disease, but it is the health professional's responsibility to provide the patient and parent/caregiver the appropriate tools to overcome it.

Care pathways as defined by the AAPD are “documents designed to assist in clinical decision-making; they provide criteria regarding diagnosis and treatment and lead to recommended courses of action” [41]. The care pathways described below are summarized in Table 3.

**Table 3:** Care pathways for caries management based upon risk assessment for ages 0–6 years.

**Risk category**	**Diagnostic**		**Preventive interventions**				**Restoration**
	**Periodic oral exams**	**Radiographs**	**Fluoride**	**Diet counseling**	**Self-management goals**	**Sealants**	**Existing lesions**
**CARE PATHWAYS FOR CARIES MANAGEMENT BASED ON RISK FOR CHILDREN 0-6 YEARS OF AGE**							
Low	6–12 mos	12–24 mos	Brush twice daily with F toothpaste^¥^	No	No	No	
Moderate	6 mos	6–12 mos	Brush twice daily with F toothpaste^¥^ optimize F intake^£^ FV every 6 mos	Yes	Yes	On enamel defects and pits & fissures at-risk	Active surveillance for developing lesions
High	3 mos	6 mos	Brush twice daily with F toothpaste^¥^ optimize F intake^£^ FV every 3 mos	Yes	Yes	On enamel defects and pits & fissures at-risk	Remineralize enamel-only lesions with FV; restoration of cavitated lesions, or non-surgical caries management with ITR or SDF as appropriate.
Very high: with extensive existing disease	Monthly	6 mos	Brush three times daily with F toothpaste^¥^ optimize F intake^£^ FV every 1–3 mos Consider additional therapies for caries control^*^	Yes	Yes	All pits and fissures	Consider caries control prior to surgical tx. Remineralize enamel-only lesions with FV; restoration of cavitated lesions, or non-surgical caries management with ITR or SDF as appropriate

¥ *Smear of 1,000 ppm fluoride toothpaste for 0–2 year-olds, pea-size of fluoride toothpaste for 3–6 year-olds (or equivalent for specific area)*.

£ *Recommend drinking fluoridated water (from tap or bottled), parental brushing, spit and don't rinse toothpaste*.

* *Wipe with baking soda/xylitol, use casein phosphopeptide–amorphous calcium phosphate (ACP/CPP) paste*.

*FV, fluoride varnish; ITR, interim therapeutic restoration; SDF, silver diamine fluoride; mos, months*.

#### Low Caries Risk Management Protocol

If the plaque levels are low as an indication of adequate home care, and fluoride exposure has prevented signs of disease under their current dietary conditions, patients should be praised and advised to continue their daily routine. Chemical therapy indicated for infants and toddlers, namely in the form of fluoride toothpaste at least twice daily, must be included in the treatment plan for all patients (even low risk) [37] in the appropriate amount. The AAPD and the ADA recommend a smear or an amount the size of a grain of rice for children 0–2 years, and pea size for 3–6 years when using a 1,000 ppm F toothpaste [38, 39], as it is likely to be sufficient to maintain a healthy caries balance in low-risk patients. Fluoride-free “training toothpaste” should not be recommended as its use has not proven to have the same therapeutic effect as fluoride toothpaste. Recalls for periodic re-evaluation should be set for every 6 months, where their preventive home care routine should be reinforced. Low risk patients do not benefit from in-office fluoride applications [53, 54]. Radiographic examinations, if necessary (contact areas closed and not visible) and feasible (if patient's cooperation allows, and according to local regulations) should be performed at 12–24 month intervals as per AAPD and ADA guidelines [55, 56].

#### Moderate Caries Risk Management Plan

Even with no signs of caries lesions at any stage, moderate risk children will present with several risk factors that indicate a greater chance of developing caries in the near future and that additional chemical therapy could prevent frequent acid exposure from tipping the balance to the establishment of disease. Caregivers and children (when appropriate) should be informed about the caries process and counseled on strategies to improve their individual dietary or home care routines. Anticipatory guidance should be provided, as described above. Fluoride toothpaste recommendations indicated above should be stressed, additional forms of fluoride exposure (fluoride in drinking water) should be promoted, and children at moderate risk should be recalled at 6-month intervals for monitoring of adherence to the improvement of diet and home care routines. These patients will also benefit from in-office FV applications at 6-month intervals starting at the first visit. Radiographic examinations should be performed every 6–12 months.

#### High Caries Risk Management Plan

Children with obvious signs of caries at any stage and children with several risk factors and minimal fluoride exposure, are at high risk of developing more lesions in the future. In addition to the chemical therapy (F toothpaste recommendations and promotion of other forms of fluoride exposure as well as use of agents that enhance remineralization, acid neutralization, or inhibit MS transmission), and behavioral counseling to improve practices as mentioned above, patients at high risk benefit from additional in-office FV applications at 3–6 months intervals. Therefore, 3–6 month recall visits should include FV application, reinforcing self-management goals to reduce specific risk factors, promote protective factors and perform active surveillance of lesions at all stages.

The caries management plan should include a restorative treatment plan that aims to limit tissue destruction, diminish sensitivity to allow adequate plaque control measures and restore function and form, taking into consideration the cooperation and health status of the patient, as well as the family situation. Following principles of minimal intervention dentistry [21], the choice of restorative treatment (which is typically needed in high risk patients), could include traditional restorative treatment or non-surgical therapies [interim therapeutic restorations with glass ionomer cements, caries arrest with silver diamine fluoride (SDF), etc.] after careful discussion explaining to the parents the risk and benefits of each option, and trying to delay or defer more complicated and risky procedures like sedation and/or general anesthesia. The informed consent of the parent is essential following this discussion and laying out of recommended options.

#### Very High Risk Patients With Extensive Treatment Needs-Additional Guiding Principles

The outcomes studies described above [17] and the results of our 6 year through adult studies [12] show that in-office topical fluoride applications and home fluoride toothpaste use may not be sufficient to prevent future caries in high-risk patients. When there is a prolonged acidic environment in the plaque created by frequent sugary/carbohydrate diet and poor oral hygiene this leads to microbial dysbiosis and serves as the driving force for caries formation in children [26, 57] resulting in high caries recurrence in high risk children [51, 58, 59]. Therefore, home care behavior modification can be the key to caries management in children.

Children at high risk, who already require extensive restorative treatment (for example, more than four restorations), may benefit from intensive care including preventive sealants in surfaces “at risk.” As studies show that supervised brushing achieves much higher prevention results than brushing alone [42, 43], supervised brushing with a fluoride toothpaste should be a major point in the counseling sessions. Brushing three times a day (after every meal) and spitting the toothpaste with no rinsing [60] are simple strategies that may maximize the protective action of fluoride on these children.

Additional possible antimicrobial regimens to consider are wiping/brushing teeth with xylitol [61–63] and/or baking soda [64–66] after feedings or meals. Xylitol is non-cariogenic, and baking soda is an effective acid naturalizing agent, which can effectively neutralize the oral environment and have antiplaque and antimicrobial effects in children and adults [64–66].

For children with numerous cavitated lesions who may need multiple visits to complete restorative care and/or may have limited cooperation for treatment, SDF therapy can be used to achieve caries arrest and desensitization of lesions with no pulpal involvement. Sensitivity from open lesions can be a significant barrier for implementation of effective plaque removal, creating a vicious circle that can easily be broken by doing initial caries control by arresting and desensitizing lesions with SDF or glass ionomers depending on the location and visibility of the lesions and preference of the parents. Once better homecare has been established, and less sensitivity is followed by improved behavior, plaque retentive lesions can be followed-up at subsequent visits and if necessary, restored with glass ionomer cement interim restorations to prevent plaque accumulation and combined with FV at 3-month intervals to prevent new lesions [67–70]. This combination therapy can help to delay or defer more complicated and risky procedures like sedation or treatment under general anesthesia, which is especially important for children under 3 years of age.

The care pathways for caries management for each of the assessed caries risk levels for ages 0–6 years are summarized in Table 3.

#### Conclusions

Successful management of dental caries in young children requires a risk-based approach to formulate an individualized treatment plan using a chronic disease management model, which aims at targeting the patient's specific risk factors (biological, environmental and social) that contribute to the establishment and progression of this multifactorial disease with adequate education, support and follow-up to guide the patient to sustained health outcomes.

### Caries Risk Assessment–Practical Step-By-Step Guidelines for the Age Group 6 Years Through Adult

Parts of the following sections are reproduced and updated from Featherstone et al. [4] with permission.

The following are step-by-step guidelines for use of the CAMBRA CRA system with the age group 6 years through adult. Details are given in the following sections. The CAMBRA system identifies four caries risk levels, namely low, moderate, high and extreme. CRA takes place as part of the regular comprehensive oral exam in the following sequence, leading to formulating an individualized caries management treatment plan that includes chemical therapy. Here are the steps in the process:

1. Evaluate dental and medical history.
2. Evaluate prevention items with the patient and ask questions that provide answers for biological and environmental risk factors in the CRA form (Table 2). Enter the answers into the CRA form or the electronic version. This can all be done by a dental assistant, dental hygienist, or equivalent.
3. Conduct clinical examination. Detect caries lesions early enough to reverse or prevent progression.
4. Assess and document the caries risk as low, moderate, high or extreme utilizing data from 1, 2, 3 above and the short list of questions listed in the CRA form (Table 2).
5. Produce and document a treatment plan that includes caries management, chemical therapy and necessary restorative treatment appropriate to the caries risk level.
6. Prescribe and/or provide chemical therapy for the patient, that includes fluoride with or without antibacterial therapy, based upon the caries risk level.
7. Use minimally invasive restorative procedures, if necessary, to conserve tooth structure and function.
8. Recall and review at intervals appropriate to the caries risk level.
9. Reassess and document caries risk level at recall and modify the treatment plan as necessary.

The first 4 steps of the process comprise the CRA, which identifies, protective factors, biological and environmental risk factors, and clinical status to provide an individualized, overall portrait of caries risk (as per Table 2). In the following steps, that CRA, in turn, informs the development and implementation of a personalized caries management plan as described in detail below. Hence, CAMBRA is a two-phase process involving both CRA and management of caries as a biologically determined, clinical disease. Steps 1, 2 and 3 are familiar elements of any conventional oral examination and form the basis of the CRA. Step 3 provides a list of what are called “disease indicators,” which are simply clinical signs of the presence of caries, most likely ongoing over time.

Step 4 uses a few simple questions (as listed in the CRA form in Table 2) to attempt to identify the potential causes of the ongoing disease, or to evaluate whether it is under control. Only those factors that have been shown to be statistically significantly related to ongoing caries risk or reversal are included here [18]. Table 2 is a ready to use CRA form. Definitions of terms follow here, and instructions are provided in the Table 2, part 2.

#### Protective Factors (Table 2, Column 1)

Protective factors are environmental factors, biological factors or chemical therapy that helps to swing the caries balance to caries prevention or reversal. The most important factors that are proven effective for CRA are:

1. Lives, goes to school, or works in a ***fluoridated drinking water area***
2. Uses a ***fluoride toothpaste at least once daily***
3. Uses a ***fluoride toothpaste at least twice daily*. **It is well-established that twice daily provides considerable added benefit [42, 43]. If the patient provides a yes to this question, a yes should also be marked to item 2
4. Uses a ***high concentration prescription (5,000 ppm F) fluoride toothpaste*** twice daily
5. Has had ***FV*** applied in the last 6 months
6. Uses ***0.05% sodium fluoride mouthrinse*** daily
7. Uses ***0.12% chlorhexidine gluconate mouthrinse*** daily for 1 week each month as prescribed for caries control, or other proven antibacterial treatment [50]
8. Has ***adequate salivary flow and function*** by inspection or measurement

Each of these items with a positive response receives a “yes” score. Yes scores in this category reduce the level of risk.

Note: xylitol use is no longer listed as a protective factor in this revised CRA version as the evidence is limited [46]. For patients with high frequency carbohydrate consumption, xylitol gum or lozenges can be recommended. Chewing a sugar free gum enhances saliva flow and thereby provides additional protection.

#### Biological and Environmental Risk Factors (Table 2, Column 2)

The following are biological and environmental risk factors that have been shown to be statistically related to caries risk [12, 18]:

1. ***Frequent snacking on fermentable carbohydrates***, at least three times daily outside of mealtimes. Frequent snacking on fermentable carbohydrates is a major caries risk factor. Snacking on fermentable carbohydrates more than 3 times daily between meals is the minimum for this risk factor. Snack foods that contain fermentable carbohydrates are those that contain, or are comprised of, glucose, sucrose, fructose, high fructose corn syrup, cooked starch. It includes juices such as apple juice and sticky fruits such as raisins.
2. Use of ***medications that induce hyposalivation***. Xerostomia is a side effect of some of the most commonly prescribed medications, and risk of dry mouth increases with the number of medications prescribed [32]. Medications in the classes of antianxiety, antidepressants, antihistamines, and antipsychotic can have hyposalivatory side effects, depending on the individual's reaction. Multiple hyposalivatory medications are much more likely than one to have a measurable effect on salivary flow and function. Examination of the medical/dental history will highlight the use of these medications if they are present. These medications may be the reason that a patient has severe tooth decay.
3. Daily, or regular ***use of recreational drugs***. A simple yes answer in this category does not indicate what drugs are in use. It is a red flag, however. Not all drugs are hyposalivatory, however, hard drugs have severe hyposalivatory effects, such as methamphetamine. “Meth mouth,” caused by methamphetamine use, is serious rampant decay with major destruction of the teeth. This is an example of extreme caries risk.
4. ***Heavy plaque on the teeth*. **This observation is a straightforward clinical observation where the practitioner simply observes that “there is heavy plaque on the teeth.” There is no specific quadrant, nor selection of teeth, nor calibrated amount. This simple measure, as observed by the clinician, has been shown in our clinical outcomes studies in thousands of patients to be a strong indicator of cariogenic bacterial activity, and it is strongly related to ongoing caries [12, 17, 18, 25]. Note: at the time of writing there is no validated chairside test commercially available for measuring cariogenic bacterial levels so this item from earlier CRA versions is no longer included in the current CRA.
5. ***Reduced salivary function (hyposalivation) as assessed by observation or by*** ***measurement*. **Hyposalivation is extremely serious to the oral health of the patient. Reduction in all of the beneficial components of saliva is serious and can lead to rampant and severe dental caries, which will become more serious over time and is very difficult to control. Hyposalivation, together with other high caries risk factors, signals extreme caries risk. The clinical signs of hyposalivation are: lack of saliva, difficulty stimulating salivary flow, dull and non-glistening soft tissue surfaces, patient complains of “dry mouth.” The stimulated saliva flow rate can be measured easily at chair side. The patient is asked to chew on sugar-free gum and spit continually into a small measuring cup for 3 min. At the end of 3 min measure the ml of saliva produced, divide by 3 and the result is ml/minute of saliva flow. More than 1.0 ml/min is normal, and <0.5 ml/min is hyposalivatory.
6. ***Deep pits and fissures*. **Deep pits and fissures are developmentally present in some teeth, provide traps for plaque and potentially put these sites at higher risk for the action of cariogenic bacteria over time. The application of sealants is appropriate.
7. ***Exposed tooth roots***. Most patients over the age of 35 years have exposed tooth roots. Gingival recession with age leads to more root surface exposure, that is a necessary, but not sufficient condition for root caries. Exposed roots do not cause root caries and most patients live with exposed roots and no decay. When coupled with other high risk caries factors the patient is predisposed to root caries. In those cases, preventive fluoride therapy is essential.
8. ***Orthodontic appliances***. Orthodontic appliances, especially bonded stainless steel brackets, are sites for cariogenic bacteria to grow and thrive preferentially, at the border between the enamel and the bracket. Smooth surface decay (white spots) around the brackets occurs in a high percentage of orthodontic patients. This is dental caries caused by cariogenic bacteria that remain present after the brackets are removed and predispose the patient to further decay. Orthodontic appliances lead to preferential growth of cariogenic bacteria during the time of the orthodontic treatment [71].

In the risk assessment procedure, any items on this list with a positive response are marked with a yes (Table 2) in the appropriate column. Each yes in columns 2 and 3 adds to the risk level. Items 1 and 4 under biological or environmental risk factors above can be modified by behavioral management. A yes to item 5, reduced salivary function, indicates extreme risk if other risk factors and disease indicators suggest at least high risk. Deep pits and fissures suggest the use of preventive sealants (depending on the age and risk status of the patient). Item 3, the use of recreational drugs, most likely indicates hyposalivation, depending on the drugs used. However, this needs to be confirmed by clinical observation or clinical measurement. Older people almost all have exposed tooth roots, indicating more attention is needed to fluoride and other preventive measures. Orthodontic appliances, such as brackets, place the patient at least into moderate risk.

#### Disease Indicators (Table 2, Column 3)

Disease indicators are the clinically observed results of previous and/or ongoing dental caries destruction of the tooth mineral.

1. Observed ***cavitation*** or radiographic evidence of progression into the dentin
2. ***White spot lesions*** (that are new or active) on smooth surfaces
3. Radiographic evidence of ***non-cavitated demineralization into the enamel*** (usually by bitewing radiographs)
4. ***Existing restorations*** placed due to caries in the last 3 years for a new patient, or in the last year for a patient of record. A new patient becomes a patient of record after the first visit, necessary restorations are completed, and from then on, the 1 year rule applies for any new restorations.

For a new patient visit, one or more of the first three disease indicators usually signals “high caries risk.” For a patient of record at a follow up visit any new appearance of any of the above disease indicators usually signals at least “high caries risk.” For example, however, a patient who is using all of the recommended chemical therapy and has been doing so for some time and has no new caries lesions the risk may be considered moderate risk.

If ***hyposalivation*** is present, in addition to disease indicators and other high risk factors (see below), this usually signals “extreme risk.”

#### Determining the Caries Risk as Low, Moderate, High or Extreme

When the CAMBRA CRA form is completed (Table 2) the health care provider makes a final determination of the caries risk level. Instructions for determining the risk level are provided in Table 2, part 2. The determination of low, moderate, high or extreme risk is guided by visualizing the balance among protective factors, risk factors and disease indicators. To aid in this visualization we have included a simple quantitative aid known as CAMBRA123. Protective factors in column 1 that are marked yes each receive a score of −1. Risk factors in column 2 with a yes are each scored +2. Yes answers to disease indicators in column 3 each receive a score of +3. Then simply add the scores for columns 2 and 3 and subtract the total from column 1. Consult the chart in Table 2, part 2 and be guided to a caries risk level. The yes indications are also used to modify behavior or determine additional therapy (see below).

### Caries Management Based on Risk Assessment- Practical Step-By-Step Guidelines for the Age Group 6 Years Through Adult

The following are step-by-step guidelines for use of the CAMBRA system for caries management for children 6 years and older and adults. Parts of the following sections are reproduced and updated from Featherstone et al. [4] with permission.

1. Carry out a CRA as described above and classify the patient as low, moderate, high or extreme caries risk.
2. Produce and document a caries management plan that addresses all the risk factors that may contribute to the development or progression of disease for that specific patient, including behavior modification for caregivers/child or adult to achieve plaque control and diet improvements.
3. Prescribe and/or provide chemical therapy for the patient, that includes fluoride with or without antibacterial therapy, based upon the caries risk level and the age of the patient. Details are described below. Consider integrating motivational interviewing (MI) principles with caregivers and patients (when age appropriate) to set up achievable goals for home management plans [20, 21].
4. Use minimally invasive restorative treatment (if needed) to conserve tooth structure and function.
5. Recall and review at intervals appropriate to the caries risk status.
6. Reassess and document caries risk level at recall and modify the treatment plan as necessary.

CAMBRA therapies for older children and adults place special importance on chemical therapy, because placing restorations can restore tooth form and function but does not affect the risk factors that caused the disease, such as a cariogenic diet or high levels of cariogenic bacteria in the mouth [50–52]. One antimicrobial chemical therapy that has been shown effective as part of a caries management plan in children 6 years and older and in adults is chlorhexidine mouthrinse [6, 12, 50, 72].

#### Chemical Therapy Needed According to the Caries Risk Assessment

The following guidelines have been used and proven by a practice–based clinical trial and by outcomes assessment in thousands of patients [6, 12]. Chemical therapy, such as fluoride toothpaste, must be included in the treatment plan for all patients (even low risk) [37]. Fluoride-containing agents are likely to be sufficient to maintain a healthy caries balance in low-risk or moderate-risk patients. Restorative treatment, as needed will be included, in conjunction with the chemical therapy. The restorative treatment which is typically needed in high risk patients, must be done according to the principles of minimally invasive dentistry [73]. The biggest issue related to success of the CAMBRA treatment is adherence to the chemical therapy, especially when it is home-use. It is essential to work with the patient through MI and counseling so that they use the home use regimens as prescribed, or the therapy will not be effective.

#### Low Caries Risk Chemical Therapy

The guideline is to “keep it simple.” Whatever the patient is doing appears to be working. If the plaque levels are low, oral hygiene looks good, and the patient uses a fluoride toothpaste at least twice daily, then the recommendation is simple: “keep doing what you are doing and use an over the counter fluoride toothpaste (1,000–1,450 ppm F) at least twice daily.” Recall for a follow up visit at 12 month intervals.

#### Moderate Caries Risk Chemical Therapy

The moderate caries risk patients need additional therapy to keep them at this risk level, or even better, to move them to low caries risk. Two alternatives are given, depending on the patient's willingness and motivation to adhere to an ongoing homecare regimen.

1. Alternative 1: Over the counter fluoride toothpaste twice daily, plus 0.05% sodium fluoride (220 ppm F) mouthrinse daily at night. The patient should also be counseled to reduce between meal snacking, and to follow this regimen conscientiously.
2. Alternative 2: Prescription, high fluoride (5,000 ppm F) toothpaste, at least twice daily, plus counseling on reducing between meal snacking of fermentable carbohydrates (substituting with xylitol-containing lozenges or candies). This regimen is very simple and is recommended for those who may not be willing or motivated to use a nightly fluoride mouthrinse. The disadvantage is the need to prescribe the fluoride toothpaste and the additional cost, or the non-availability of the 5,000 ppm F toothpaste. The advantage of this second alternative is the simplicity of the protocol, leading to better likelihood of adherence.

Recall at 6 monthly intervals for follow up visits.

#### High Caries Risk Chemical Therapy

The high-risk patient MUST have ***antibacterial therapy*** to lower the bacterial challenge. Fluoride alone, at whatever concentration and frequency, will not be enough and caries lesions will continue to develop. The best proven antibacterial therapy that we currently have available is chlorhexidine mouthrinse (or gel). There have been numerous clinical studies that have investigated the role of chlorhexidine in caries control over several decades. Early studies showed considerable promise [74–76]. However, most of the studies looked at chlorhexidine as a single agent to control caries and the results have been mixed as described in a literature review by Autio-Gold [77], in which it was concluded that “chlorhexidine rinses, gels and varnishes or combinations of these items with fluoride have variable effects.” The diversity of chlorhexidine regimens and delivery systems makes comparison very difficult amongst all the reported studies. Since the Autio-Gold publication [77] two clinical trials have been reported that have demonstrated considerable success with high caries risk patients when chlorhexidine is used in combination with fluoride therapy as part of an overall caries management plan, provided a specific chlorhexidine timed regimen is used [6, 50]. The chlorhexidine regimen reported in those studies [6, 50] is the one that is recommended below for high caries risk patients in the 6 year through adult age group.

New and better therapy will be available in the future. SDF has recently gained popularity, and guidelines for use in children and adolescents including those with special health care needs have been published [69]. There are several systematic reviews on SDF [67, 68, 70] that confirm its efficacy for arresting cavitated lesions as well as the arrest and prevention of root caries in the elderly. However, SDF has severe staining as a side effect and its use requires specific conversations around the expected staining in order to gain informed consent prior to placement. Hypochlorite (bleach) based antibacterial caries rinse is also marketed, but at the time of writing there is no published clinical trial demonstrating its efficacy and there may be safety concerns for use in children.

As of the time of writing, the following is the proven management strategy for high caries risk patients [12, 50]. There are three components:

a) FV applied in the clinic at the time of the clinical visit and reapplied every 4–6 months (for children and adults)
b) Brushing with a prescription, high fluoride (5,000 ppm F) toothpaste, at least twice daily, plus counseling on reducing between meal snacking of fermentable carbohydrates.
c) Rinse for 1 minute once daily for 1 week each month with a chlorhexidine gluconate mouthrinse (0.12%) [50]. This should be done at least 1 h apart from the fluoride tooth brushing, preferably last thing at night before bed. The regimen is to be continued for at least a year, until the disease is controlled as shown by a lower the risk level and no new clinical signs of caries.

Recall at 4–6 month intervals for follow up visits.

#### Extreme Caries Risk Chemical Therapy

The extreme risk patient MUST have antibacterial therapy to lower the bacterial challenge. Fluoride alone, at whatever concentration and frequency, will not be enough and the caries will continue to develop. The management strategy is the same as for high risk (including antibacterial therapy) PLUS additional buffering.

a) FV applied in the clinic at the time of the clinical visit and reapplied every 4–6 months (for children and adults)
b) Brushing with a prescription, high fluoride (5,000 ppm F) toothpaste, at least twice daily, plus counseling on reducing between meal snacking of fermentable carbohydrates.
c) Rinse for 1 minute once daily for 1 week each month with 10 ml of a chlorhexidine gluconate mouthrinse (0.12%). This should be done at least 1 h apart from the fluoride tooth brushing, preferably last thing at night before bed. The regimen is to be continued for at least a year, until the disease is controlled, and the risk level is lowered to moderate or low.
d) Rinse *ad libitum* throughout the day every day with a baking soda solution, made fresh daily (2 teaspoons in 8 ounces (250 ml) of water).
e) In cases where caries lesions progress even with the above regimen consider adding the home use of fluoride trays with 5,000 ppm F gel for 5 min daily.

Recall at 3–4 monthly intervals for follow up visits.

#### High and Extreme Risk Patients—Guiding Principles

In the case of high and extreme caries risk patients, their caries progression cannot be controlled by conventional fluoride therapy and conventional restorative treatment alone. All clinical studies on such patients clearly show major caries progression in spite of combined fluoride and restorative therapy. Therefore, antibacterial therapy, dietary modification, fluoride therapy and minimally invasive restorative procedures must all be used in combination to manage dental caries in high and extreme risk patients. In extreme risk patients pH control must also be added, as described above. In cases where patients do not appear to be responding and caries lesions progress, additional therapy may be needed, such as home use fluoride gel, additional antibacterial therapy such as SDF, and additional help to assist the patient with adherence.

### Implementation of Caries Management in a Clinical Practice Setting for All Age Groups—Patient and Practice Commitments

Implementing the CAMBRA system delivers to dental practices, institutions, health policy makers and educational establishments a new capability to manage caries and influence patient behavior. This capability not only applies to dental practices, but also to the potential for interprofessional collaboration (especially for the 0–6 age group) so that pediatricians and primary care providers can evaluate oral health, educate parents and families, and refer children to dental homes. While the CAMBRA system involves changing patients' mindsets and attitudes, it may involve changing dental team members' mindsets and attitudes, as well. With training and coaching, support and encouragement, dental staff members can learn how to interview patients effectively using motivational interviewing (MI) skills and gain self-satisfaction using them. They can learn how to assist patients in setting self-management goals and achieving them. They can build on their skills in delivering oral health education tailored to patients' oral health literacy levels. It is important to note however that MI may not work well in some populations and other approaches relevant to the region, ethnic group, cultural entity, etc., may be necessary to help with patient compliance. A critical part of the success of the CAMBRA approach is having the patients utilize the home-use regimens correctly. Local health care workers will need to determine what methods will work for them and their patients.

Implementing CAMBRA into practice goes smoother when the whole team is engaged, kept informed and is able and encouraged to give input and feedback. Making decisions as democratically as possible helps to keep the whole team invested. Decisions principally involve how to incorporate CAMBRA into the workflow. CAMBRA does add time to the patient visit and this requires scheduling adjustments. Whether the additional time is significant, or nominal depends upon the dental team members' communication proficiency and time management skills. With training and experience, both improve over time.

The questions on the caries risk assessment form are asked in open-ended fashion using MI, or other culturally relevant tactics. MI is a way of creating effective dialogue with patients so patients will share genuinely their health behaviors [78, 79]. Open-ended questions require more time, thought, and effort for patients to answer, but they elicit helpful insights. Sometimes ambivalence to making health behavior changes surfaces. MI guides patients through their ambivalence. The interviewer's affirmations are designed to empower patients by helping them to recognize their intrinsic strengths. The interviewer's reflective listening allows patients to clarify misinterpretations and add more depth to their responses. Summaries by the interviewer are a way of pulling together the information gathered during the CRA in order to guide patients toward action.

The benefits of taking time to perform the CRA using MI skills are that patients are more likely to take self-responsibility and make sustainable health behavior changes when they select goals that they believe are important and achievable. Sometimes patients prefer to break goals into incremental steps; in such cases, progress is monitored at each patient encounter.

A prepared outline for each type of CAMBRA visit (initial, recall, treatment) and standard talking points promote visit consistency for all patients. Scripting patient education helps to keep the visit on track, but scripting must also allow for differences in patients' oral health literacy levels. With attention to time management, the added visit length does not detract from overall practice productivity. When all clinical staff members are trained on the CAMBRA system, any available staff members can be deployed to perform parts of the CAMBRA component of the patient visit.

In the course of CAMBRA visits, staff will invariably encounter patients who will struggle to make changes and adhere to their caries self-management goals, however. With coaching, dental staff members can learn how to help patients who have low self-efficacy, that is, little confidence in their ability to make changes. On-line videos and continuing dental education courses/webinars can assist with didactic training in coaching techniques.

Another key decision relates to how the therapeutic products will be made available to the patients. Options include writing prescriptions. If the patients will receive prescriptions, the dental staff will need to make sure the selected pharmacy actually stocks the products. Another option is to dispense the products at the practice, either by selling them on a retail basis or on a fee basis (in the US using CDT code D9630). The option to make the products available gratis, although very generous, does not necessarily lead to a commitment by the patients to use them. Even if the practice does not want to charge full price, a nominal fee reinforces the notion to the patient that the products have value. If the CAMBRA therapeutics are dispensed at the practice, dental staff will need to find the time and space to maintain the inventory and follow rules for dispensing the prescription drugs.

**Figure 1:**
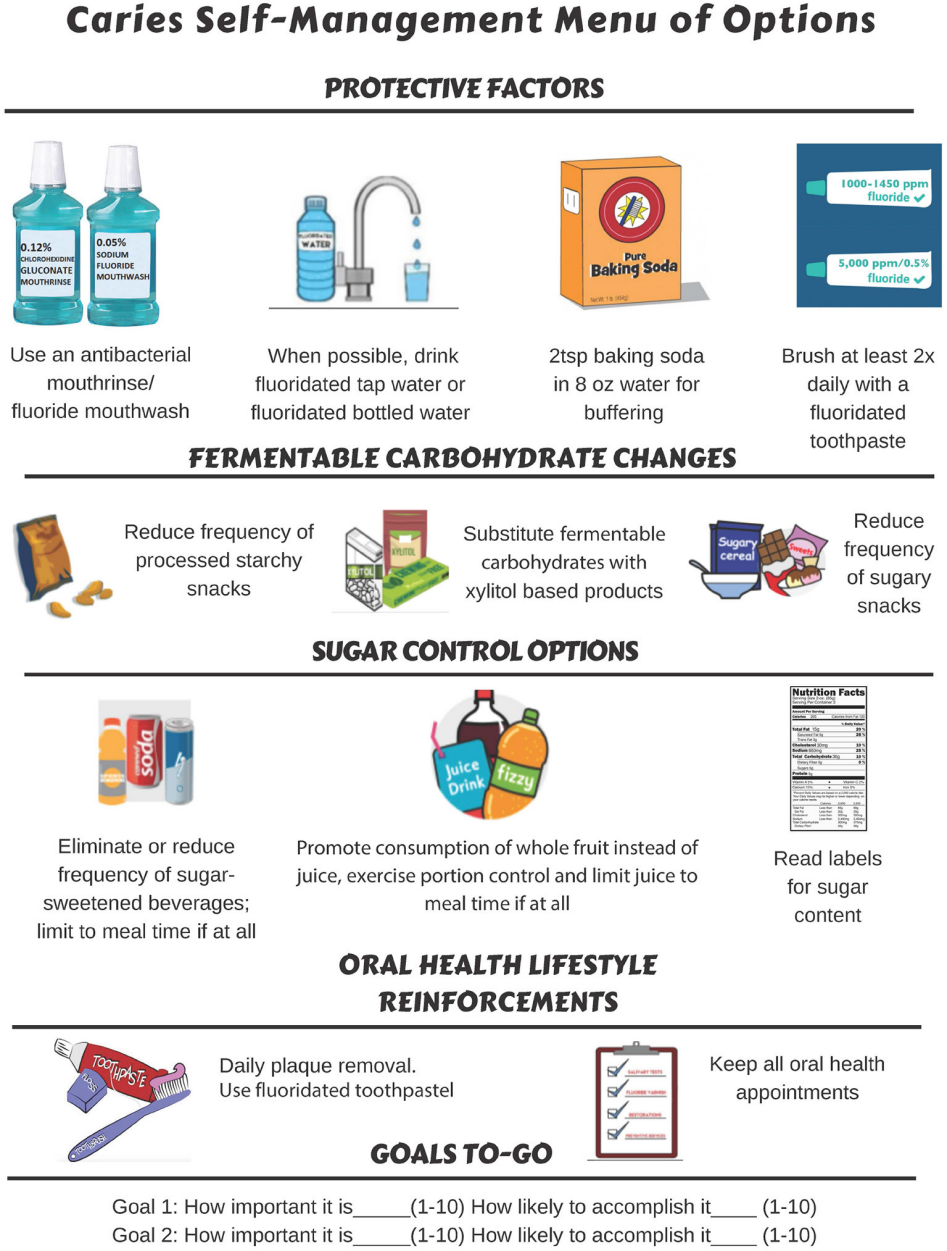
Caries self-management menu of options.

Taking care to tailor the delivery of information to patients' oral health literacy levels improves patient understanding. A concise written summary of patients' self-management goals is helpful for post-visit recall. Figure 1 is an example of a check sheet that can be used to assist patients to determine their specific goals.

Although the entire dental team is involved, dental practices may benefit from having a CAMBRA champion helping to drive the implementation process. The CAMBRA champion may be a dentist, dental assistant, dental hygienist, or dental care coordinator. The CAMBRA champion will identify resources, such as CAMBRA webinars, On-line videos, continuing dental education courses, arrange Lunch and Learn meetings, speak to dental supply representatives about new products, will function as a troubleshooter, and keep the team motivated. It behooves the CAMBRA champion to take the time to check-in with staff during staff meetings and informally. The CAMBRA champion should stay sufficiently attentive to the clinic environment to identify opportunities and barriers proactively to long-term sustainability of the CAMBRA system in the practice.

When patients understand caries as a chronic disease and adhere to their personalized caries self-management plans, the behavioral changes they make are likely to be more sustainable. They are more motivated to keep their appointments and complete their treatment plans. They do not want to face re-care due to failure to manage the aspects of caries disease that are within their control. The reward for staff is satisfaction in successfully providing high quality, evidence-based, patient-focused successful dental care.

One limitation of the approach presented in this document is that even though the evidence base was derived from experience in California in academic, public and private practice settings the results may not be fully applicable in all cultures, countries or for all races and ethnicities. However, the San Francisco Bay Area has one of the most diverse populations anywhere in the world and the evidence reviewed here was accumulated in many thousands of patients including white, black, Asian, Native American and from many different countries of origin.

## Conclusions

Use of a CRA form allows the clear identification of the individual risk factors that lead to the development of caries lesions, aids the clinician to establish an effective caries management plan, and empowers the patient with the knowledge to change the disease path. The evidence-based CRA forms and application directions, presented here, provide a concise handbook for dental health care providers to use in practice as an aid to determine the caries risk status of individual patients. Its consistent use over time can allow both the clinician and the patient to evaluate the impact and success of the preventive and treatment interventions during the course of treatment.

The CAMBRA caries management guidelines presented can be used as a handbook to aid the care provider in making patient-centered decisions for successful caries management. The steps for implementing CAMBRA in a practice foster the establishment of a partnership for health with the patients to fight this multifactorial disease with adequate education, support and follow-up, to guide them to sustained health outcomes.

## Author Contributions

JF, YC, PA, BC, SD, PR, LZ, and FR-G contributed to the planning, writing, scientific and clinical content, reviewing, and editing of this document. All authors contributed to the article and approved the submitted version.

## Conflict of Interest

The authors declare that the research was conducted in the absence of any commercial or financial relationships that could be construed as a potential conflict of interest.
